# Circular RNA *circ0007360* Attenuates Gastric Cancer Progression by Altering the *miR-762*/*IRF7* Axis

**DOI:** 10.3389/fcell.2022.789073

**Published:** 2022-02-17

**Authors:** Yawei Xing, Hongxia Chen, Zixiang Guo, Xiaodong Zhou

**Affiliations:** ^1^ Department of Gastroenterology, The First Affiliated Hospital of Nanchang University, Nanchang, China; ^2^ Department of Gynecology and Obstetrics, The First Affiliated Hospital of Nanchang University, Nanchang, China

**Keywords:** circ0007360, gastric cancer, progression, miR-762, IRF7

## Abstract

Gastric cancer is a major health burden worldwide. Circular RNAs (circRNAs) are a novel family of noncoding RNAs that are involved in multiple types of cancers, including gastric cancer. As biological functions and the underlying molecular mechanisms of the newly identified circRNA *circ0007360* have not been investigated, our present study focused on the role of *circ0007360* in the progression of gastric cancer. After characterizing *circ0007360* as a cytoplasmic circRNA, we revealed the inhibitory effects of *circ0007260* on the survival, migration, invasion, and stemness of gastric cancer cells. Subsequently, *miR-762* was identified as a direct target microRNA (miRNA) of *circ0007360* and was proved to act as a vital downstream transcript to fulfill the tumor-promoting effects in the absence of *circ0007360*. Furthermore, we demonstrated that interferon regulatory factor 7 (*IRF7*), which was validated as a target gene of *miR-762*, serves as an indirect target of *circ0007360* to attenuate the progression of gastric cancer. Moreover, *in vivo* experiments confirmed the potentiation of gastric cancer cell growth and stemness upon depletion of *circ0007360*. In summary, our results revealed that activation of the *circ0007360/miR-762/IRF7* axis is a novel mechanism for the attenuation of gastric cancer progression. Our study unveils the diagnostic and therapeutic values of *circ0007360* in patients with gastric cancer.

## Introduction

Gastric cancer, which has adenocarcinoma as a major subtype (accounting for 90% of all cases), is caused by multiple clinical factors. On the one hand, *Helicobacter pylori* infection is usually considered as an initial stimulus for the tumorigenesis of gastric cancer (Correa, 2013). On the other hand, mutations in inflammatory response genes have been shown to strongly correlate with the initiation of gastric cancer by facilitating the colonization of bacteria (Persson et al., 2011). For example, polymorphisms in genes, such as inflammatory *IL1B*, are associated with the risk of gastric cancer (Camargo et al., 2006). Because of late diagnosis, the 5-year survival rate of patients with gastric cancer is less than 20% (Anderson et al., 2010). Therefore, developing effective biomarkers for diagnosis and prognosis is of great worth for patients with gastric cancer.

Cancer stem-like cells (CSCs) not only contribute to resistance to chemotherapeutic strategies but also act as a pivotal cell population for the metastasis and recurrence of cancer, including gastric cancer (Takaishi et al., 2009; Garcia-Mayea et al., 2020). Thus, targeting gastric CSCs, which are characterized as a CD44^+^ population, holds dramatic promise for treating patients with gastric cancer (Bekaii-Saab and El-Rayes, 2017).

Circular RNAs (circRNAs) are a class of noncoding RNAs that form an exonuclease-resistant loop by direct back splicing (Eger et al., 2018). Although some circRNAs are nuclear RNAs, most of them have been demonstrated to localize in the cytoplasm and to serve as sponges for microRNAs (miRNAs) by matching miRNA binding sites (Salzman, 2016; Vo et al., 2019). As a subgroup of small RNAs, miRNAs are well-characterized as posttranscriptional regulators of target messenger RNAs (mRNAs) by binding to their 3′ untranslated regions (3′UTRs) (Matsuyama and Suzuki, 2020). Both circRNAs and miRNAs play vital roles in cancer progression (Goodall and Wickramasinghe, 2021). For instance, circRNA *circNRIP1* is capable of negatively regulating the levels of *miR-149-5p* in gastric cancer cells and thereby potentiating AKT1/mTOR pathway to trigger cancer progression (Zhang et al., 2019).

Since *circ0007360* (we also named this circRNA as circular RNA number 4779; *circ4779*) is an ill-investigated circRNA, hereby we focused on studying the biological effects of *circ0007360* on the survival, migration, invasion, and stemness of gastric cancer cells and the underlying molecular mechanism. First, a couple of classical assays were performed to validate the circular characteristic of *circ0007360* and its cytoplasmic localization. Subsequently, *miR-762* was identified as a downstream miRNA target of *circ0007360*. Importantly, the absence of *miR-762* mitigated the enhancement of cell survival, migration, invasion, and stemness, which was induced by the depletion of *circ0007360*. Furthermore, we identified interferon regulatory factor 7 (*IRF7*) as a target gene of *miR-762* and performed a series of rescue experiments to validate that *IRF7* is an indirect target of *circ0007360* to exert its tumor-suppressive role. Importantly, *in vivo* experiments further consolidated the inhibitory effects of *circ0007360* on gastric cancer cell growth and stemness. Our investigation may aid in the development of *circ0007360* as a promising prognostic marker and as a therapeutic candidate for the treatment of gastric cancer.

## Material and Methods

### Cell Culture and Treatment

Human gastric cancer cell lines AGS and MKN-7 were purchased from IMMOCELL (Xiamen, Fujian, China). Both cell lines were maintained in Eagle’s minimum essential medium supplemented with 10% fetal bovine serum, 100 U/ml penicillin, and 100 U/ml streptomycin. All the reagents were purchased from Gibco. Actinomycin D (1 μM; Sigma) was used to check the stability of RNA transcripts by treating cells for the indicated time points.

### RNA Fluorescent *In Situ* Hybridization (FISH)

To check the localization and expression of *circ0007360*, we used the RNA FISH method. The whole procedure was performed according to the instructions from the Fluorescent *in situ* Hybridization Kit (Rabobio; C10910). The probes were also purchased from Rabobio as follows: FISH Probe Mix (Red; Rabobio; C10920), h-*U6* FISH Probe Mix (Red) (Rabobio; LNC110101), and h-*18S* FISH probe mix (red; Rabobio; LNC110201).

### gDNA Extraction

Total gDNA was isolated from AGS cells using the DNeasy Blood and Tissue Kit (Qiagen; 69504). Divergent and convergent primers were used for polymerase chain reaction (PCR) amplification of *circ007360* and *GAPDH*, respectively. The PCR products from cDNA or gDNA were separated using agarose gel electrophoresis. All primers used for the PCR are listed in Supplementary Table S1.

### Cloning

The *circ0007360* ectopic expression construct was generated using PCR amplification and ligated to the pCDNA3.1 backbone. *Circ0007360* and *IRF7* shRNA were generated by inserting the annealed shRNA oligos into the pLKO.1-TRC backbone. The reporter constructs for wild type *circ0007360*, mutant *circ0007360*, wild type *IRF7* 3′UTR, or mutant *IRF7* 3′UTR were amplified and inserted into the pmirGLO (Promega) backbone. All primers used for cloning are listed in Supplementary Table S1.

### Real-Time Quantitative PCR (RT-qPCR)

Total RNA was isolated using the Total RNA Extraction Kit (Vazyme Biotech, Nanjing; China). Subsequently, 1 μg of RNA was reverse transcribed using the HiScript II First Strand cDNA Synthesis Kit (Vazyme Biotech). Next, the cDNA was subjected to quantitative PCR using the ChamQ SYBR Master Mix kit (Vazyme Biotech) and iQ5 qPCR machine (Bio-Rad) to detect the relative expression levels of target genes. The relative levels of various target genes were quantified using the 2^−ΔΔCt^ formula, with *18S* RNA as the reference transcript for normalization. All primers used in this study are listed in Supplementary Table S1. RNase R treatment was performed as follows: 1 μg RNA was diluted in 40 μl water supplemented with 4 U RNase R (Thermo Fisher), after which the mixture was incubated for 15 min at 37°C.

### Subcellular Fractionation

To detect the localization of *circ0007360*, the PARIS^™^ Kit (Thermo Fisher; AM 1921) was used to extract total RNA from the cytoplasm or nucleus. RT-qPCR was performed as described above.

### Transfection

Cells were plated in wells of six-well plates and transfected with 4 μg plasmids or 200 pmol miRNA mimics or inhibitors using Lipofectamine 3000 (Invitrogen) according to the manufacturer’s instructions.

### Luciferase Reporter Assay

AGS cells were co-transfected with the indicated luciferase reporter plasmids and the *miR-762* mimic or mimic NC. At 48 h post-transfection, the cells were lysed and luciferase activity was measured using the Dual-Glo Luciferase Assay System (Promega). Firefly luciferase activity was normalized to Renilla luciferase activity.

### MTT Assay

Briefly, 24 h after transfection, the cells were plated at 1 × 10^3^ cells/well in 96-well plates. MTT solution (20 μl, 5 mg/ml; Qiancheng Biotech, China) dissolved in PBS was directly added to each well at the indicated time points. After incubation for 4 h at 37°C, the medium was replaced with 100 μl DMSO, and the absorbance at 490 nm was measured using a SpectraMax Absorbance Reader (Molecular Devices). The results are presented as the mean ± SD (*n* = 6).

### Colony Formation Assay

At 24 h post-transfection, 500 cells were plated in the wells of a six-well plate. After culturing for 2 weeks at 37°C, the cells were fixed with 4% paraformaldehyde for 10 min. Staining was performed using 0.5% crystal violet for 10 min. The stained cell colonies were photographed and counted after three washes with water.

### Flow Cytometry Assay

To quantify the frequencies of cells in different cell cycle phases, at 24 h post-transfection, the cells were fixed with 70% ethanol overnight at 4°C. Subsequently, the cells were washed twice with PBS before permeabilization in PBS buffer supplemented with Triton X-100 (0.2%) and RNase (10 μg/ml) for 30 min. Subsequently, the cells were stained with propidium iodide (PI; 20 μg/ml; A211-02; Vazyme Biotech) solution in the dark for 30 min at room temperature (RT), after which 1 × 10^4^ cells were quantified using a NovoCyte flow cytometer (ACEA Biosciences, San Diego, CA, United States).

Similarly, the cell apoptosis assay was initiated by treating the transfected cells with Annexin V-fluorescein isothiocyanate (200 μg/ml) and PI (30 μg/ml) in the dark for 10 min at RT. After several washes with PBS buffer, the NovoCyte flow cytometer was used to determine the frequency of apoptotic cells.

To check the expression of CD44 and EpCAM on the cell surface, cells were stained with isotope-FITC, anti-CD44-FITC, or anti-EpCAM-FITC (all from BioLegend) and the signal was detected using a NovoCyte flow cytometer. In all the assays mentioned above, unstained cells served as negative controls to establish background fluorescence thresholds.

### Transwell Assay

For migration assays, at 24 h post-transfection, 5 × 10^5^ serum-starved cells were seeded into the upper chambers of 24-well Transwell plates (Corning, NY, United States). For the evaluation of cell invasion, a diluted Matrigel (BD) pre-coated membrane of the top chamber was used. For both assays, 10% serum medium was added to the bottom wells. After 24 h of incubation, 4% PFA was applied to fix the migrated or invasive cells on the bottom part of the chamber, and the cells were stained with 0.5% toluidine blue before imaging. Five independent fields were selected for statistical analysis.

### Tumor Sphere Formation Assay

Upon 24 h transfection, cells were trypsinized and 1 × 10^3^ cells were seeded into wells of 24-well plates containing DMEM/F12 medium (IMMOCELL, China) supplemented with B27 (Gibco), 20 ng/ml EGF (Gibco), 20 ng/ml bFGF (Nowoprotein, China), and 4 μg/ml insulin (Gibco). Fresh medium was added every 3 days and the cells were cultured for 12–18 days before being photographed.

### Western Blotting

RIPA buffer (Beyotime) supplemented with 1 × complete protease inhibitor cocktail (Roche) was used to lyse the cells. Protein concentrations were determined using a bicinchoninic acid protein assay kit (Thermo Fisher). Equal amounts of proteins were separated using SDS-PAGE and subsequently transferred onto a 45-μm polyvinylidene difluoride (PVDF) membrane (Bio-Rad). Next, the membranes were blocked with 5% non-fat dry milk in Tris-buffered saline with 0.1% Tween 20 (TBST) for 1 h at RT. The resulting membranes were probed with primary antibodies overnight at 4°C. After three washes with TBST, the membranes were incubated with horseradish peroxidase (HRP)-conjugated secondary antibodies for 2 h at RT. The signal was detected using Clarity^™^ Western ECL Substrate (Thermo Fisher) and the ChemiDoc Imaging System (Bio-Rad). All antibodies used in this study are listed in Supplementary Table S2.

### Animal Experiments

All mouse experiments were conducted in accordance with a protocol approved by the Animal Care and Use Committee of Nanchang University. For tumor growth assays, 5 × 10^6^ AGS cells with or without *circ0007360* depletion were subcutaneously injected into the lower back regions of 6-week-old female BALB/c nude mice for 34 days (*n* = 6). Tumor volumes were monitored every 4 days with calipers from 18 days post-inoculation. Tumor volumes were calculated using the following formula: length ×  width^2^/2. The mice were euthanized 34 days after inoculation, after which the tumors were collected and photographed. Moreover, the tumor weight was determined 34 days post-inoculation.

### Immunohistochemistry Analysis

First, the tumors from the mice were fixed and embedded. After sectioning, the paraffin was removed by placing the slides in xylene three times, followed by placing them in 100% ethanol twice. Endogenous peroxidase activity was blocked with 0.3% hydrogen peroxide for 20 min, after which the slides were rehydrated in 96, 70, and 50% ethanol, respectively. After antigen retrieval and washing, the slides were slowly cooled to RT, followed by three times washing with PBST. Primary antibody against Ki67 (BD; 550609) diluted (1:100) in 1% BSA solution was used to incubate the slides overnight at 4°C. The slides were washed with PBST three times before incubating with 1:200 diluted biotinylated secondary antibody (DAKO; E0353) for 30 min at RT. After three times washing with PBST, the slides were incubated with Vectastain complex (Vector Laboratories; PK-6100) for 30 min. Subsequently, the slides were washed with PBST three times and developed using the DAB reagent. Next, slides were counterstained with Mayer hematoxylin (Sigma-Aldrich; MHS80) for 45 s and dehydrated. Finally, Entellan mounting medium (Merck; 107961) was used to mount the slides. Images were captured, and the rate of positive cells was analyzed using the AIpathwell software (Servicebio, China).

### Statistical Analysis

Statistical analyses were performed using the GraphPad Prism 8 software. All results are shown as the mean ± SD. Unpaired Student’s *t*-test was used for analysis, and *p* < 0.05 was considered to be significant (*0.01 < *p* < 0.05, **0.001 < *p* < 0.01, ***0.0001 < *p* < 0.001, ****p < 0.0001). NS, not significant.

## Results

### *Circ0007360* Is a Circular RNA

**FIGURE 1:**
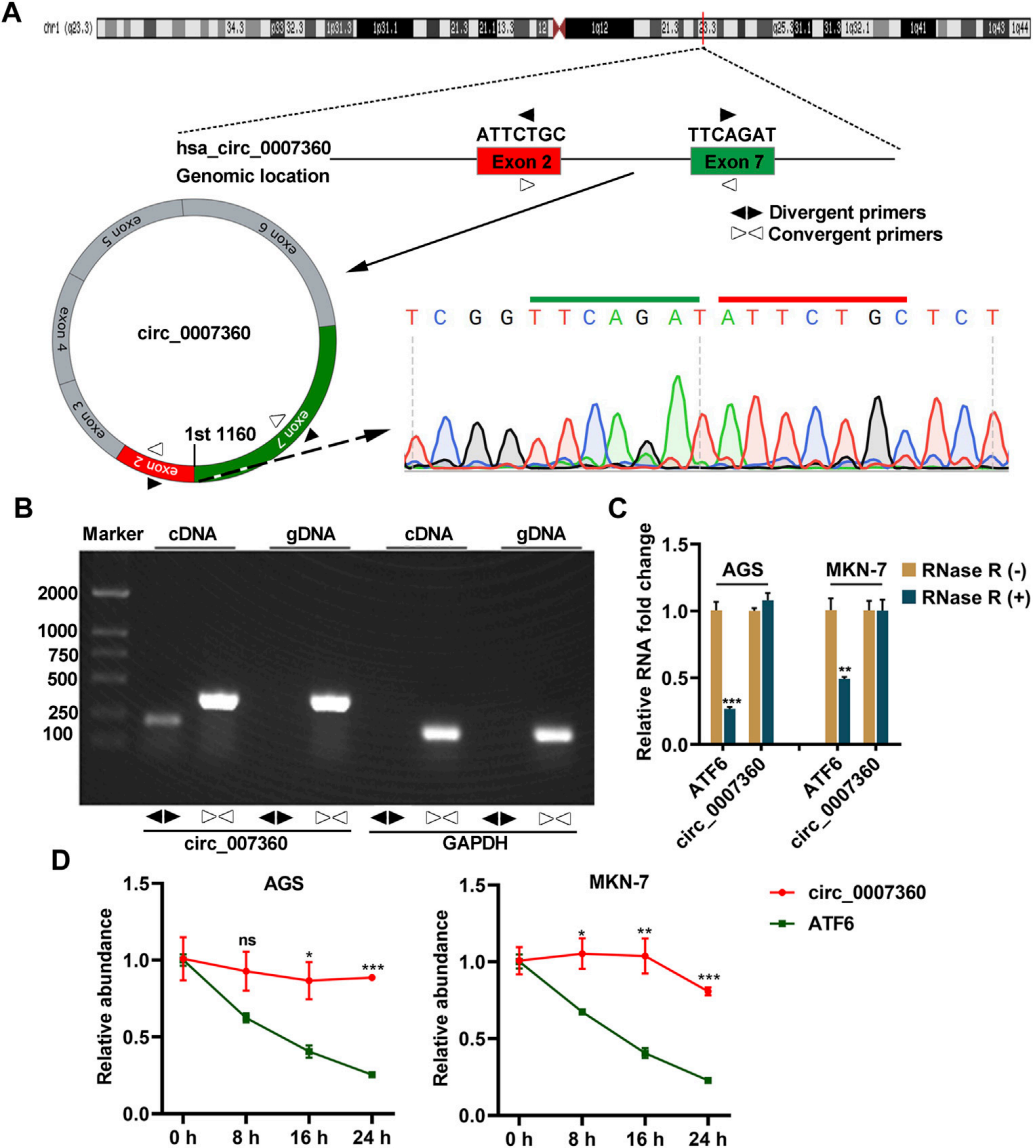
*Circ0007360* is a circular RNA. **(A)** A schematic model showing the genomic locus of *circ0007360*, the location of divergent and convergent primer pairs used for detecting *circ0007360* expression, as well as the sanger sequencing result of the junction site. **(B)** The DNA gel result for detecting the PCR products amplified by the indicated primers for *circ0007360* or *GAPDH*. **(C)** RT-qPCR quantification of the RNA levels of *ATF6* or *circ007360* with or without RNase R treatment. **(D)** RT-qPCR quantification of the RNA levels of *ATF6* or *circ007360* with or without actinomycin D treatment for the indicated time points.

We started our investigation by checking whether *circ0007360* is a real circular RNA. To this end, a convergent primer pair was used to amplify the junction site of *circ0007360*. Sanger sequencing showed that the junction site could be detected, as predicted (Figure 1A). However, as expected, the PCR products generated by the divergent primer pair could not be detected when genomic DNA (gDNA) was used as a template (Figure 1B). Of note, the convergent primer pair for *circ0007360* or *GAPDH*, which served as a positive control, amplified the expected fragment in both cDNA and gDNA (Figure 1B). Since resistance to exonuclease RNase R is also a gold standard for testing whether an RNA transcript is circular (Jeck and Sharpless, 2014), we treated the cDNA from two gastric cancer cell lines, AGS and MKN-7, with RNase R. RT-qPCR results showed that the levels of *ATF6* mRNA, which is the host mRNA of *circ0007360*, but not *circ0007360*, were reduced upon RNase R challenge (Figure 1C). Furthermore, a time-course experiment directed by actinomycin D, an inhibitor of transcription, revealed that the stability of *circ0007360* was significantly higher than *ATF6* mRNA in both gastric cancer cell lines (Figure 1D). Collectively, these results indicate that *circ0007360* is a real circular RNA.

### *Circ0007360* Is Mainly Localized in the Cytoplasm

**FIGURE 2:**
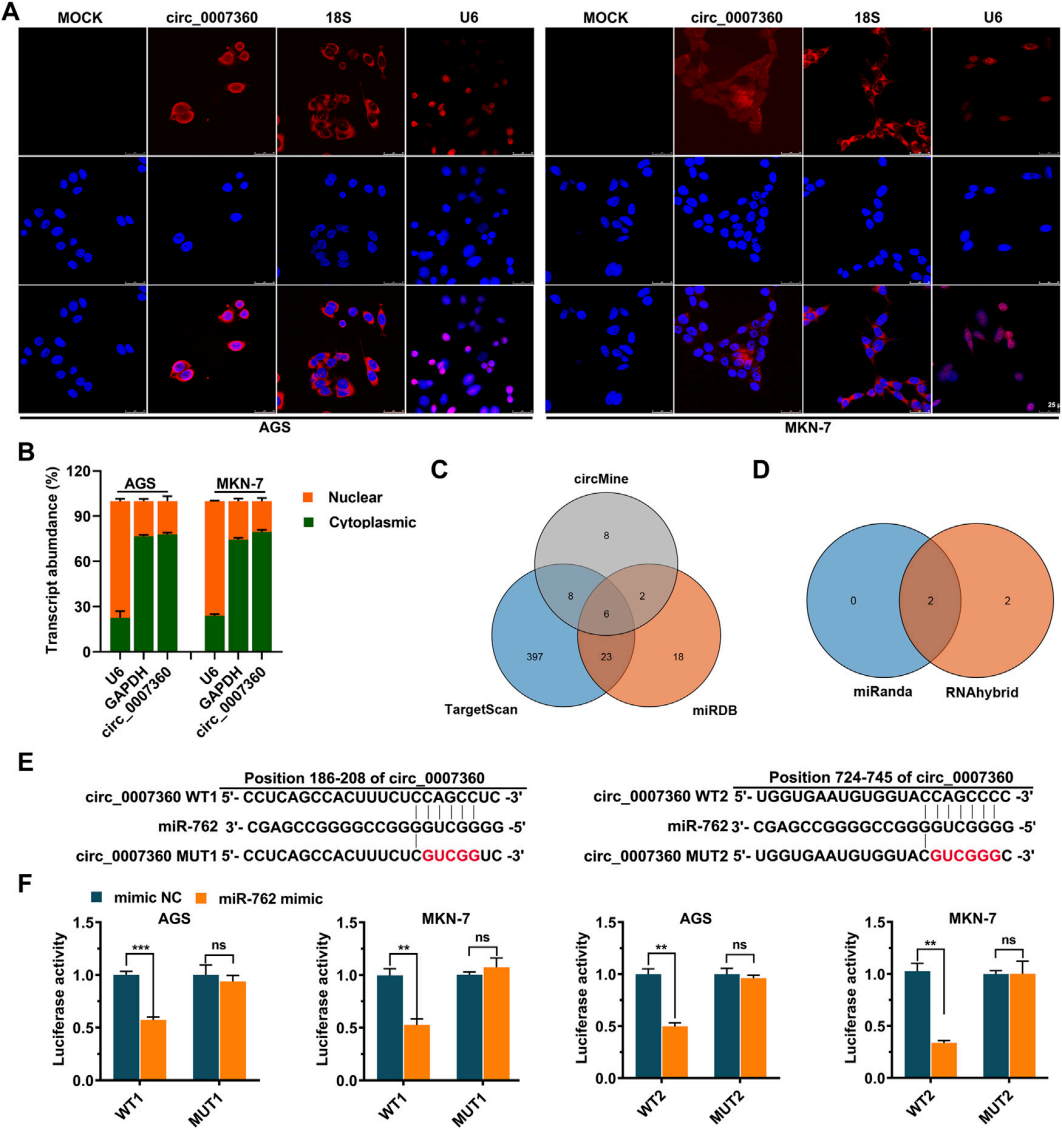
*Circ0007360* is a cytoplasmic circular RNA that may sponge *miR-762*. **(A)** Representative images from RNA fluorescent *in situ* hybridization (FISH) for detecting *circ0007360*, *18S*, or *U6* RNA. DAPI was applied to stain the nucleus. **(B)** Subcellular fractionation assay to measure the levels of *U6*, *GAPDH*, or *circ0007360* in the nucleus or cytoplasm. **(C)** Schematic plot showing the interacting site between wild type (WT) or mutant (MUT) *circ0007360* and *miR-762*. **(D)** The relative luciferase activity of WT or MUT *circ0007360* constructs in the presence or absence of *miR-762* mimic.

As the localization of circRNAs determines their functions (Hsiao et al., 2017), we next checked the localization of *circ0007360* using an RNA FISH assay. *Circ0007360* was more abundant in the cytoplasm, where the *18S* RNA was localized, than that in the nucleus, where another positive control *U6* RNA was localized (Figure 2A). Moreover, subcellular fractionation analysis confirmed the abundant cytoplasmic location of *circ0007360* (Figure 2B). Since the most common mechanism of cytosolic circRNAs is acting as sponges for matching miRNAs, we performed *in silico* prediction for target miRNAs of *circ0007360*. After analyzing the intersection result from three databases (Targetscan (Agarwal et al., 2015), miRDB (Chen and Wang, 2020) and circMine (Zhang et al., 2021)), six candidate miRNAs were enriched (*miR-762*, *miR-4427*, *miR-4656*, *miR-3135b*, *miR-4680-3p* and *miR-8077*; Figure 2C). Given the fact that *miR-762* is a well-annotated miRNA whose promoting role in cancer progression has been reported by many research groups (Li et al., 2015; Ge et al., 2019; Chen et al., 2020), we focused on *miR-762* for further experimental validation. Two putative binding sites of *miR-762* on *circ0007360* was found using the CircMir 1.0 platform (Xu et al., 2021) (Figures 2D,E). To prove the interaction between these two molecules, reporter assays were performed. We found that *miR-762* attenuated the luciferase activity of both predicted interacting fragments from *circ0007360* (WT), whereas the corresponding mutant *circ0007360* (MUT) fragments were not affected by *miR-762* overexpression (Figure 2F).

### *Circ0007360* Inhibits While *miR-762* Promotes the Survival, Migration, and Invasion of Gastric Cancer Cells

**FIGURE 3:**
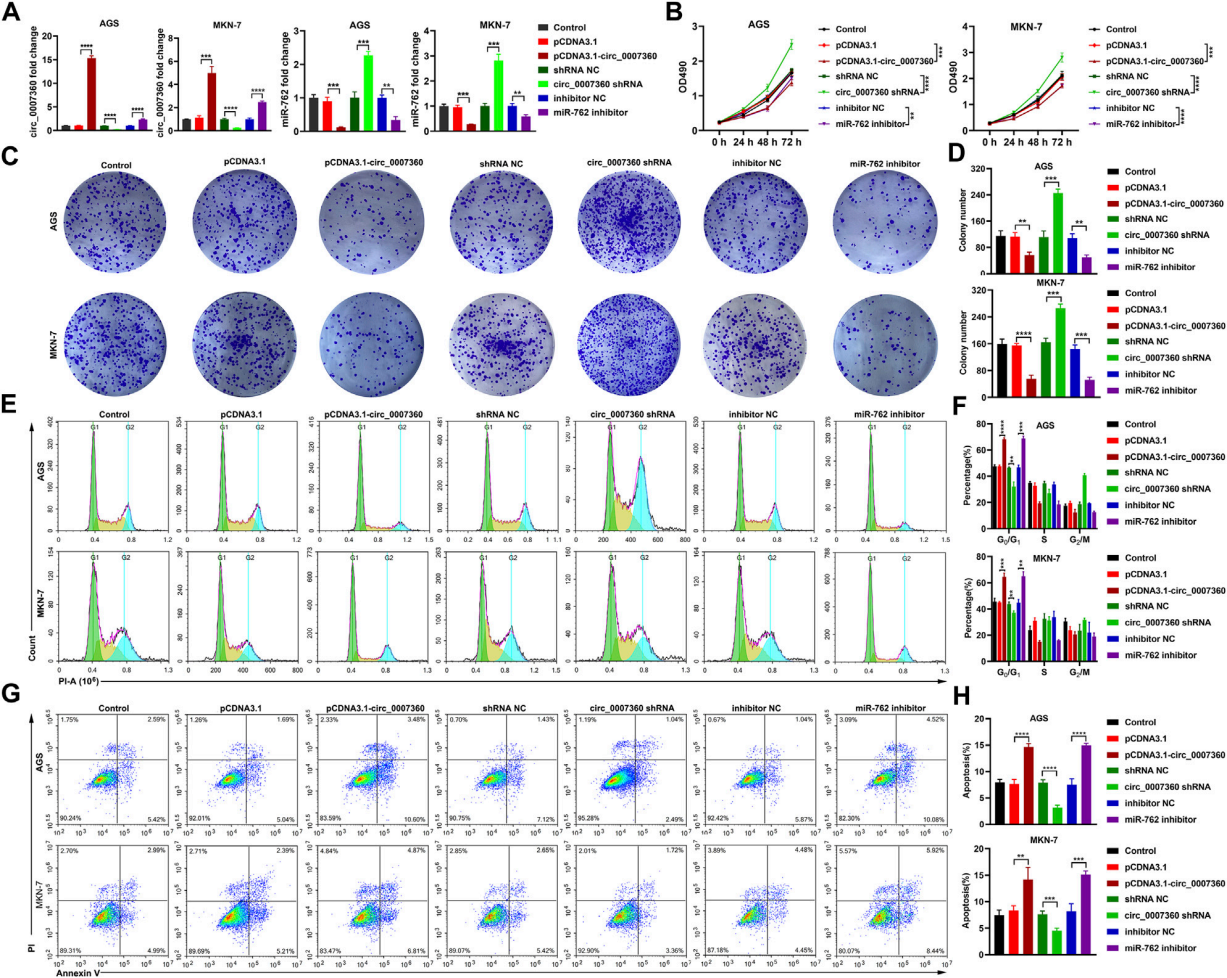
*Circ0007360* suppresses while *miR-762* enhances cell survival. RT-qPCR quantification of *circ0007360* or *miR-762* expression with ectopic expression of *circ0007360*, knockdown of *circ0007360*, or *miR-762* in AGS or MKN-7 cells. **(B)** MTT assay for detecting cell proliferation upon ectopic expression of *circ0007360*, knockdown of *circ0007360*, or *miR-762*. **(C**,**D)** Representative images **(C)** and quantification **(D)** of colony formation results from cells with ectopic expression of *circ0007360*, knockdown of *circ0007360*, or miR-762. **(E**,**F)** Representative images **(E)** and quantification **(F)** of cell cycle analysis using flow cytometry of cells with ectopic expression of *circ0007360*, knockdown of *circ0007360*, or *miR-762*. **(G**,**H)** Representative images **(G)** and quantification **(H)** of cell apoptosis analysis using flow cytometry of cells with ectopic expression of *circ0007360*, knockdown of *circ0007360* or *miR-762*.

**FIGURE 4:**
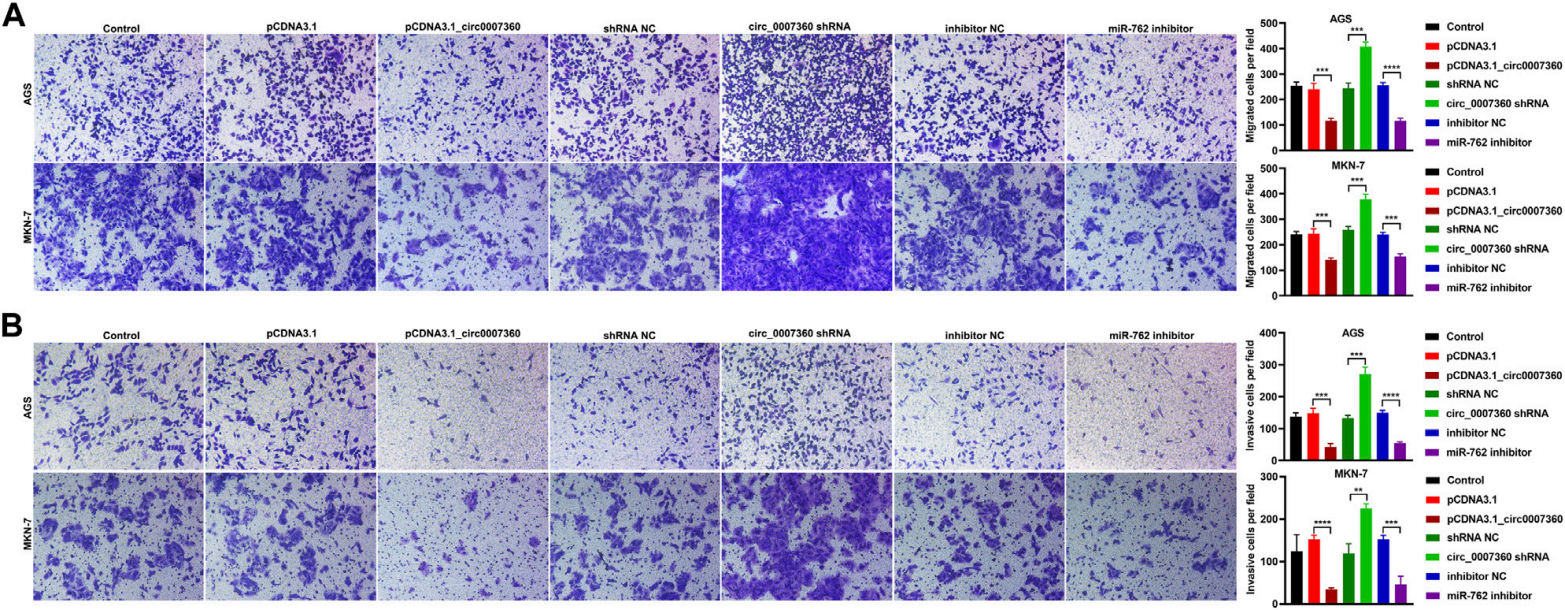
*Circ0007360* attenuates while *miR-762* augments cell migration and invasion. **(A**,**B)** Representative images and quantification of transwell results for detecting the migration **(A)** or invasion **(B)** in cells with ectopic expression of *circ0007360*, knockdown of *circ0007360,* or *miR-762*.

To investigate the effects of *circ0007360* and *miR-762* on the survival of gastric cancer cells, we misexpressed them by transfecting *circ0007360* ectopic expressing construct, shRNA against *circ0007360*, or miRNA inhibitors for *miR-762* into cells (Figure 3A). MTT evaluation suggested that overexpression of *circ0007360* or depletion of *miR-762* mitigated, while inhibition of *circ0007360* expression augmented the proliferation of gastric cancer cells (Figure 3B). Moreover, colony formation analysis confirmed the negative or positive effects of *circ0007360* and *miR-762* on gastric cancer growth, respectively (Figures 3C,D). We then tested whether the cell cycle was altered upon the misexpression of these two RNAs. Results from propidium iodide (PI)-based flow cytometry assay revealed that overexpression of *circ0007360* or knockdown of *miR-762* alleviated, whereas *circ0007360* depletion potentiated the cell cycle progression of gastric cancer cells (Figures 3E,F). In contrast, we found that the apoptosis of cells was enhanced by *circ0007360* overexpression or the absence of *miR-762* but was inhibited by silencing *circ0007360* expression (Figures 3G,H). Furthermore, we examined the migration and invasion of gastric cancer cells in the aforementioned genetic settings. Surprisingly, the migratory and invasive abilities of cells decreased significantly when *circ0007360* was ectopically expressed or *miR-762* was depleted, while the migration and invasion increased upon knockdown of *circ0007360* (Figures 4A,B). Taken together, our results demonstrate the tumor-suppressive effects of *circ0007360* and the tumor-promoting effects of *miR-762* in two gastric cancer cell lines.

### *Circ0007360* Suppresses While *miR-762* Enhances the Stemness of Gastric Cancer Cells

**FIGURE 5:**
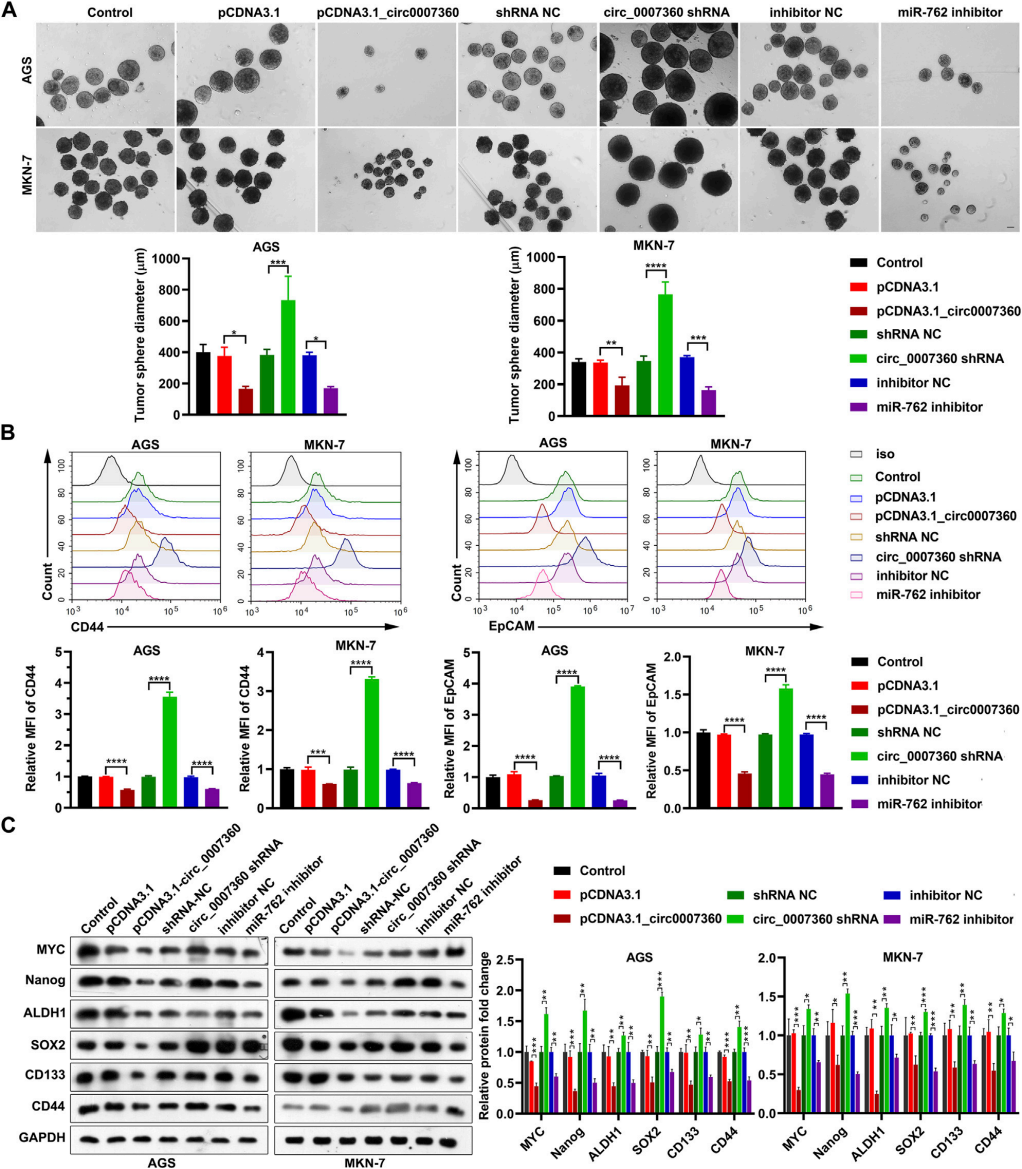
*Circ0007360* inhibits while *miR-762* promotes the stemness of gastric cells. **(A)** Representative images and quantification of tumor sphere formation results of stemness measured in cells with ectopic expression of *circ0007360*, knockdown of *circ0007360,* or *miR-762*. **(B)** Representative images and quantification of flow cytometry analysis results of the expression of CD44 and EpCAM on the cell surface in cells with ectopic expression of *circ0007360*, knockdown of *circ0007360,* or *miR-762*. **(C)** Representative images and quantification of the western blotting detection of stemness markers expressed in cells with ectopic expression of *circ0007360*, knockdown of *circ0007360,* or *miR-762*.

Considering the contribution of cancer stem cells to tumor recurrence and chemotherapy resistance (Bekaii-Saab and El-Rayes, 2017), we investigated whether *circ0007360* and *miR-762* affect the stemness of gastric cancer cells. Data from tumor sphere formation assays showed that *circ0007360* overexpression or *miR-762* depletion reduced the size of tumor spheres formed by gastric cancer cells (Figure 5A). However, the absence of *circ0007360* promoted the size of the tumor spheres (Figure 5A). We then tested the cell surface expression of stem cell markers to further evaluate the stemness of cells at the molecular level. Flow cytometry results revealed that the levels of CD44 and EpCAM were downregulated upon *circ0007360* ectopic expression or *miR-762* depletion, but the levels of the two markers were upregulated when *circ0007360* was silenced (Figure 5B). Furthermore, protein levels of other stemness markers, such as MYC, Nanog, ALDH1, SOX2, and CD133, were also reduced by the overexpression of *circ0007360* or *miR-762* depletion (Figure 5C). However, knockdown of *circ0007360* potentiated the protein levels of these markers (Figure 5C). In conclusion, *circ0007360* hinders to, while *miR-762* contributes to, the stemness of gastric cancer cells.

### The Mitigation of Gastric Cancer Progression by *circ0007360* Is Dependent on Sponging *miR-762*

**FIGURE 6:**
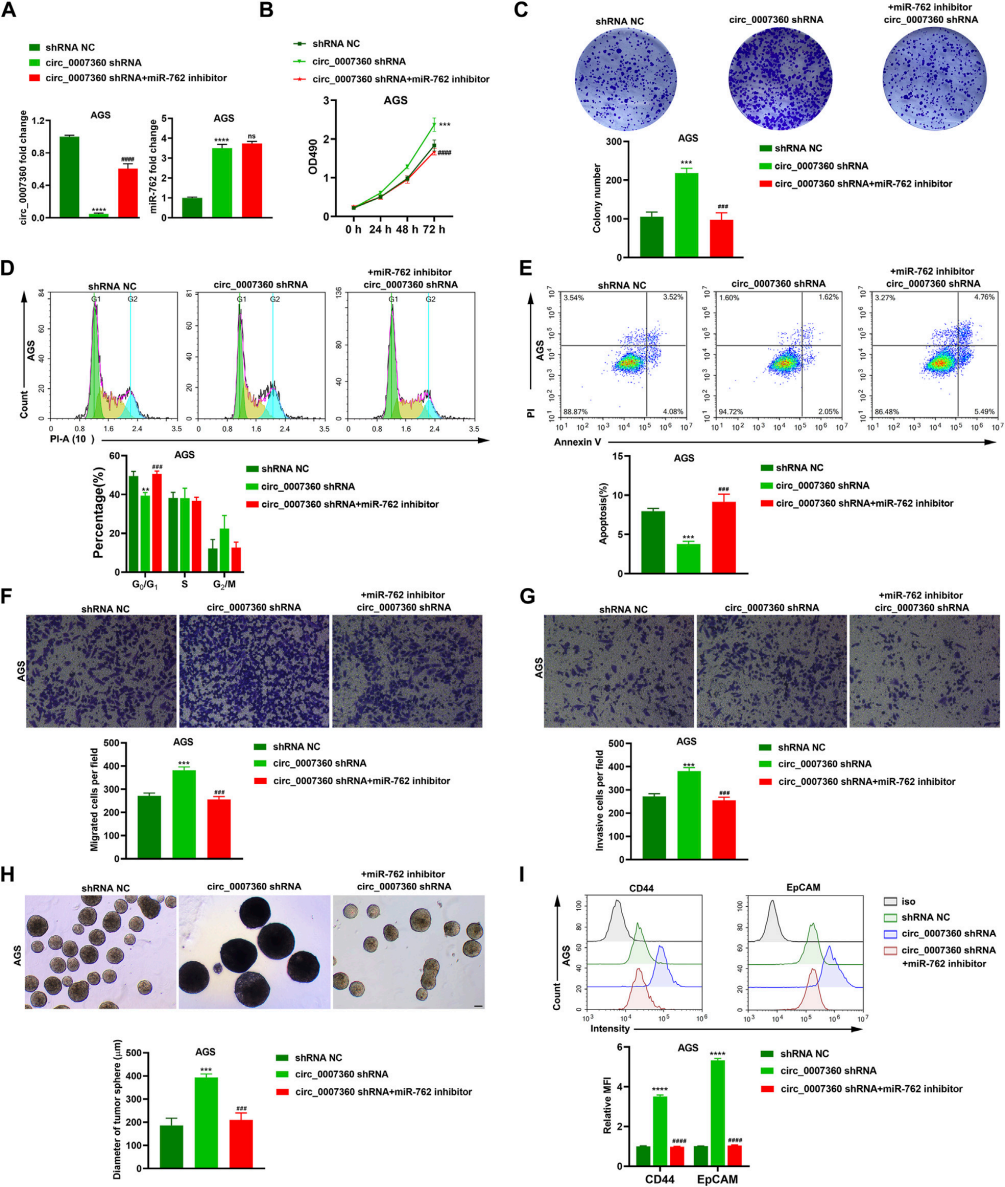
Inhibition of *miR-762* is responsible for the tumor-suppressive role of *circ0007360*. **(A)** RT-qPCR analysis of *circ0007360* or *miR-762* expression with *circ0007360* knockdown and *miR-762* re-expression in AGS cells. **(B)** MTT assay in AGS cells with *circ0007360* depletion and *miR-762* re-expression. **(C)** Representative images and quantification of colony formation assays in AGS cells with *circ0007360* knockdown and *miR-762* re-expression. **(D)** Representative images and quantification of cell cycle analysis by flow cytometry in AGS cells with *circ0007360* knockdown and *miR-762* re-expression. **(E)** Representative images and quantification of cell apoptosis analysis using flow cytometry in AGS cells with *circ0007360* knockdown and *miR-762* re-expression. **(F**,**G)** Representative images and quantification of transwell results for detecting the migration **(F)** or invasion **(G)** in AGS cells with *circ0007360* knockdown and *miR-762* re-expression. **(H)** Representative images and quantification of tumor sphere formation results of stemness measured in AGS cells with *circ0007360* knockdown and *miR-762* re-expression. **(I)** Representative images and quantification of flow cytometry analysis of the expression of CD44 and EpCAM on the cell surface in AGS cells with *circ0007360* knockdown and *miR-762* re-expression.

To validate the significance of *miR-762* in the effects exerted by *circ0007360*, we proceeded by depleting *miR-762* in gastric cancer cells with *circ0007360* knockdown (Figure 6A). Surprisingly, we observed that the enhancement of cell proliferation, colony formation abilities, cell cycle progression, cell migration, invasion, and stemness of cells, and the inhibition of cell apoptosis mediated by the absence of *circ0007360* were significantly restored when *miR-762* expression was silenced by the specific miRNA inhibitors (Figures 6A–I and Supplementary Figure S1). Taken together, the rescue experiments demonstrate that the biological effects triggered by the loss of *circ0007360* are highly dependent on the upregulation of *miR-762*, which serves as a pivotal downstream target miRNA of *circ0007360*.

### *IRF7* Is a Target mRNA of *miR-762*

**FIGURE 7:**
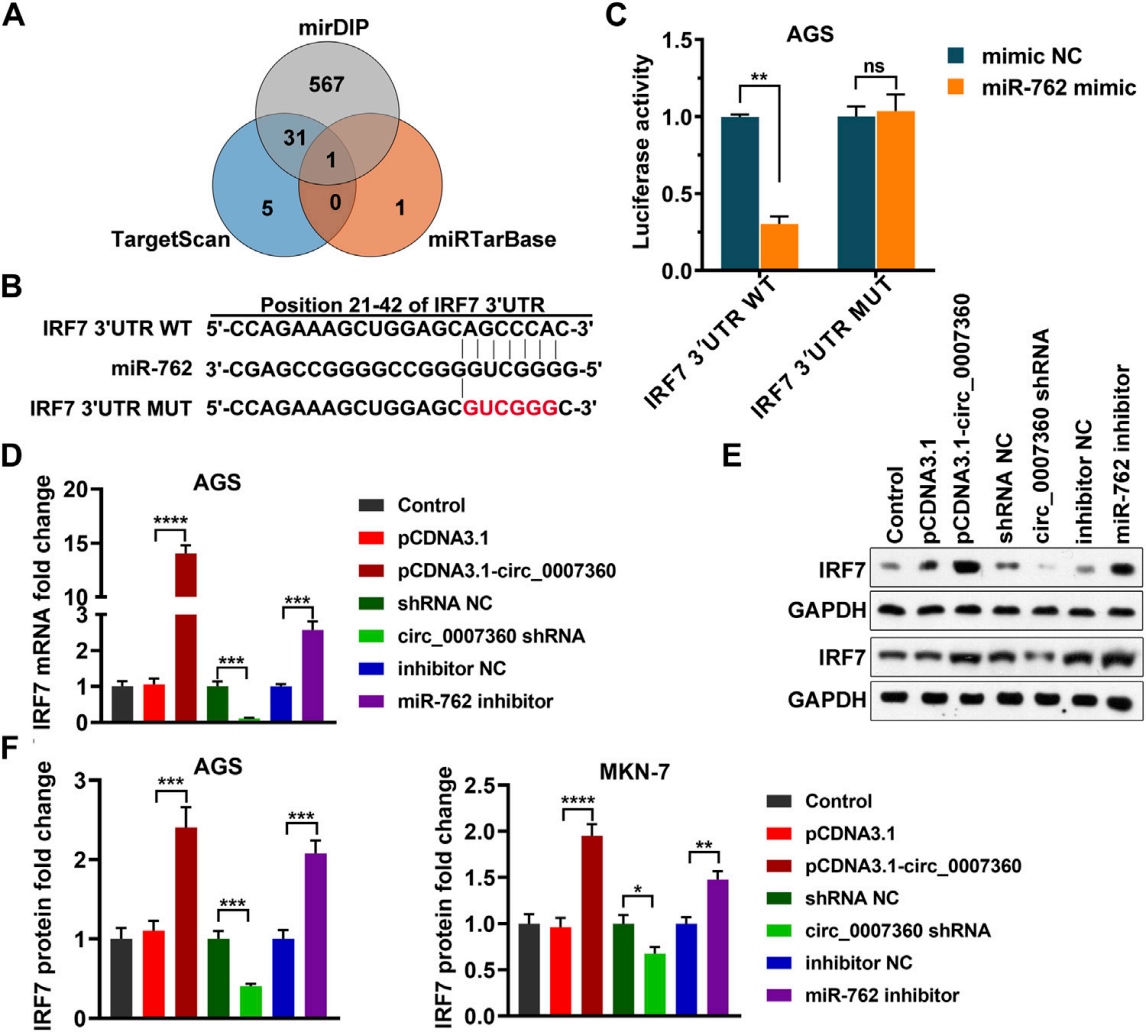
*IRF7* is a target mRNA of *miR-762*. **(A)** Venn diagram indicating the intersection between three databases for predicting the target genes of *miR-762*. **(B)** Schematic plot showing the interacting site between the wild type (WT) or mutant (MUT) *IRF7* 3′UTR and *miR-762*. **(C)** The relative luciferase activity of WT or MUT *IRF7* 3′UTR in the presence or absence of *miR-762* mimic. **(D**,**E)** RT-qPCR **(D)** or western blotting **(E)** quantification of *IRF7* expression in AGS cells with ectopic expression of *circ0007360*, knockdown of *circ0007360,* or *miR-762*. **(F)** Quantification of IRF7 protein levels in AGS or MKN-7 cells with ectopic expression of *circ0007360*, knockdown of *circ0007360,* or *miR-762*.

To screen the target mRNA of *miR-762*, we mined three well-established databases, miRDIP (Tokar et al., 2018), TargetScan (Agarwal et al., 2015), and miRTarBase (Huang et al., 2020) (Figure 7A). Of note, *IRF7* was the only mRNA hit shared by these three databases (Figures 7A,B). Next, we performed a luciferase reporter assay to validate the interaction between *miR-762* and the *IRF7* 3′UTR. As expected, *miR-762* dramatically inhibited the activity of the wild type, but not the mutant *IRF7* 3′UTR (Figure 7C). Consistently, both the mRNA and protein levels of *IRF7* increased upon depletion of *miR-762* (Figures 7D–F). Moreover, as the upstream modulator of *miR-762*, *circ0007360* positively regulated the levels of *IRF7* (Figures 7D–F). Thus, our results demonstrate that *circ0007360* may indirectly enhance the expression of *IRF7* by decreasing *miR-762* levels.

### The Augmentation of Gastric Cancer Progression Triggered by *miR-762* Is Dependent on Suppressing *IRF7*

**FIGURE 8:**
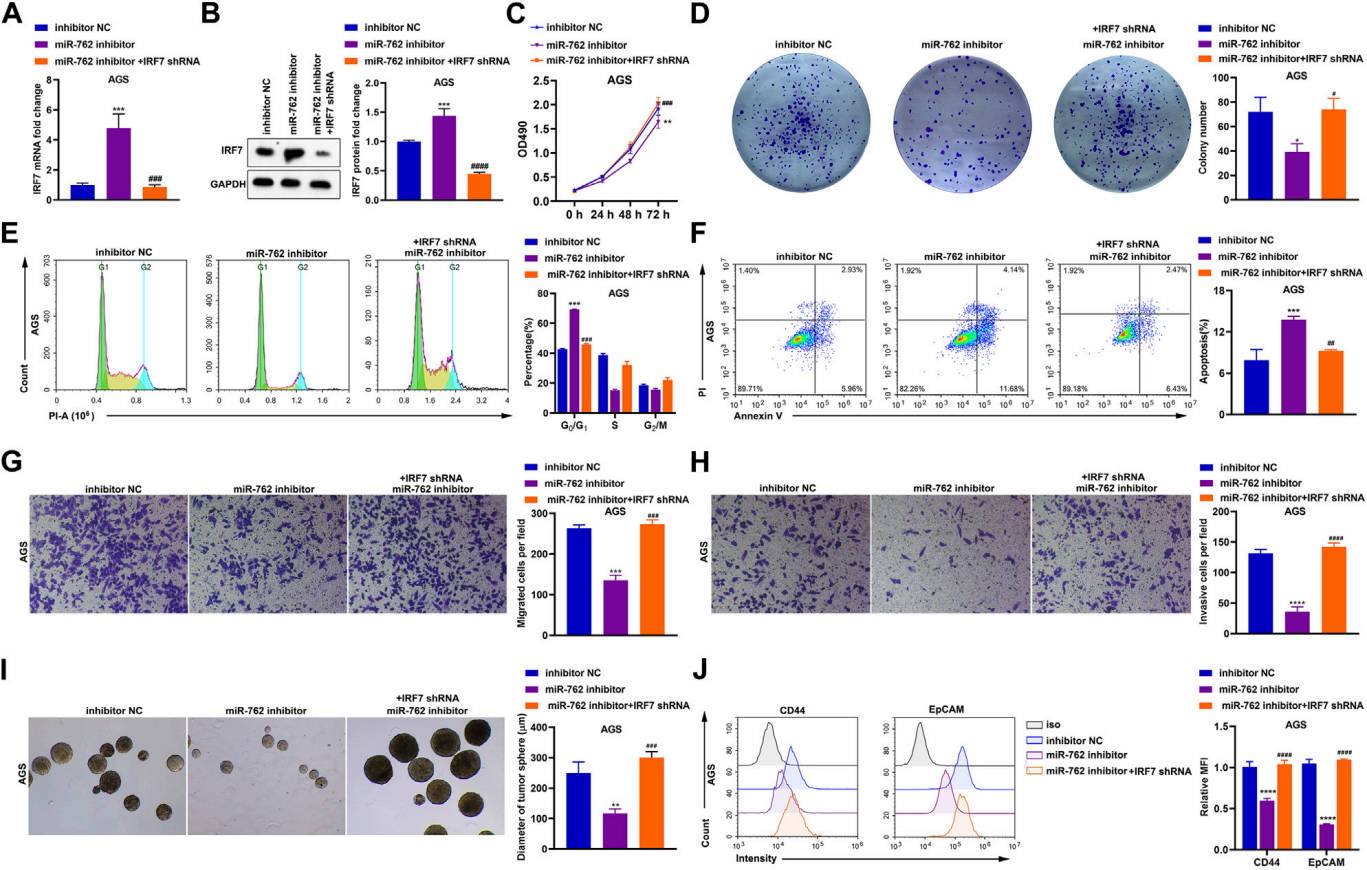
The tumor-promoting function of *miR-762* is dependent on the downregulation of *IRF7*. **(A)** RT-qPCR detection of *IRF7* mRNA expression in the presence or absence of *miR-762* inhibitor or *IRF7* shRNA. **(B)** Representative images and quantification of western blotting detection of IRF7 protein level in the presence or absence of *miR-762* inhibitor or *IRF7* shRNA. **(C)** MTT analysis in AGS cells transfected with or without *miR-762* inhibitor or *IRF7* shRNA. **(D)** Representative images and quantification of colony formation result in the presence or absence of *miR-762* inhibitor or *IRF7* shRNA. **(E)** Representative images and quantification of cell cycle analysis in the presence or absence of *miR-762* inhibitor or *IRF7* shRNA. **(F)** Representative images and quantification of apoptosis analysis in the presence or absence of *miR-762* inhibitor or *IRF7* shRNA. **(G**,**H)** Representative images and quantification of transwell analysis for checking the migration **(G)** or invasion **(H)** in the presence or absence of *miR-762* inhibitor or *IRF7* shRNA. **(I)** Representative images and quantification of tumor sphere formation in the presence or absence of *miR-762* inhibitor or *IRF7* shRNA. **(J)** Flow cytometry quantification of CD44 and EpCAM expression in the presence or absence of *miR-762* inhibitor or *IRF7* shRNA.

Since we validated that *IRF7* is a downstream target gene of *miR-762*, the following investigations were performed to confirm the significance of *IRF7* in *miR-762*-induced gastric cancer progression. First, *IRF7* was silenced by shRNA in cells depleted of *miR-762* (Figures 8A,B). Results from MTT and colony formation assays indicated that inhibition of cell survival resulting from *miR-762* knockdown was alleviated when *IRF7* was depleted (Figures 8C,D). In line with these data, the effects of *miR-762* depletion on cell cycle arrest, apoptosis, migration, invasion, and stemness were rescued upon silencing *IRF7* (Figures 8E–J). Thus, our data prove that *IRF7* inhibition is responsible for the tumor-promoting function directed by *miR-762*. Moreover, taking all these *in vitro* data together, we indicate that the progression of gastric cancer may be attenuated by the *circ0007360/miR-762/IRF7* axis.

### *Circ0007360* Suppresses Gastric Cancer Growth and Stemness *In Vivo*

**FIGURE 9:**
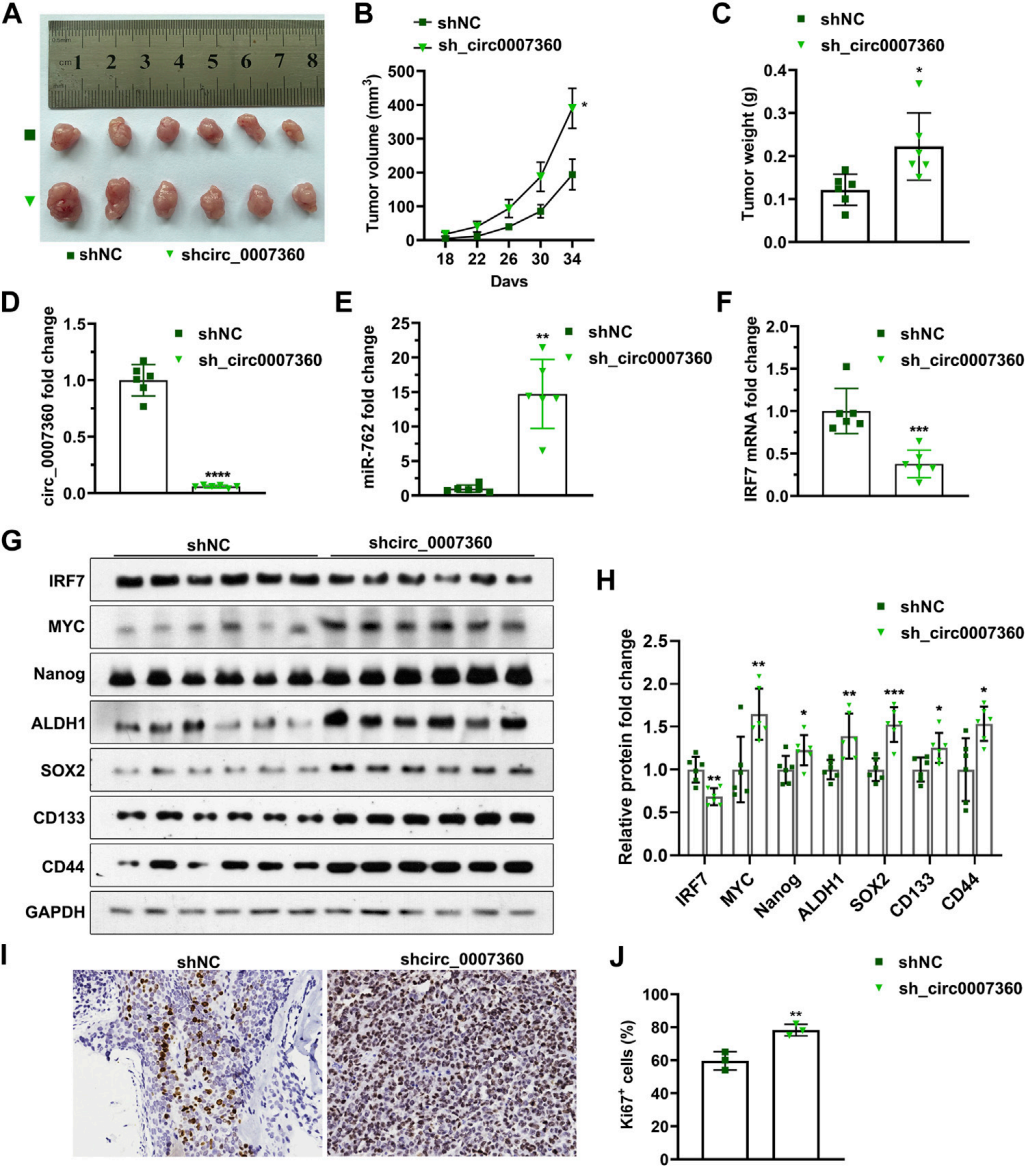
Depletion of *circ0007360* enhances gastric tumor formation in mice. **(A)** Representative images of tumors formed by cells with *circ0007360* depletion in mice *via* subcutaneous injection. **(B**,**C)** Volume of tumors **(B)** and quantification of tumor weight **(C)** formed by AGS cells with *circ0007360* depletion *via* subcutaneous injection in mice. **(D**–**F)** RT-qPCR detection of *circ0007360* **(D)**, *miR-762* **(E)**, or *IRF7* **(F)** RNA levels in tumors formed by AGS cells with *circ0007360* depletion *via* subcutaneous injection in mice. **(G**,**H)** Representative images and quantification of western blotting detection of IRF7 and markers involved in cell stemness regulation. **(I**,**J)** Representative images **(I)** and quantification **(J)** of immunohistochemistry staining for Ki67 in tumors formed by cells with *circ0007360* depletion.

To further consolidate the aforementioned *in vitro* results, we performed *in vivo* experiments to determine the effects of *circ0007360* on gastric cancer cells. Therefore, we inoculated AGS cells with or without *circ0007360* depletion in mice through subcutaneous injection and found that the tumor volume and tumor weight increased significantly in the *circ0007360* knockdown group compared with the control group (Figures 9A–C). Moreover, RT-qPCR results confirmed that *miR-762* levels were enhanced, while *IRF7* levels were reduced, upon depletion of *circ0007360* in the isolated tumors formed by the human gastric cancer cells (Figures 9D–F). Furthermore, we found that absence of *circ0007360* resulted in the upregulation of stemness markers expressed, as observed in *in vitro* assays (Figures 9G,H). Importantly, knockdown of *circ0007360* led to an increase in the percentage of Ki67-positive cells in tumors (Figures 9I,J). Collectively, the *in vivo* mouse experiments confirm the inhibitory role of *circ0007360* on tumorigenesis and cell stemness and the existence of the *circ0007360/miR-762/IRF7* axis in tumors formed in the mouse xenograft model.

## Discussion

The present study have unveiled the tumor-suppressive role of *circ0007360* in gastric cancer progression and have uncovered the modulation of the *miR-762/IRF7* axis as the underlying mechanism. *Circ0007360*, which is localized on chromosome 1, is a circRNA whose functions are yet to be investigated. Given the fact that *circ0007360* is resistant to RNase R and is relatively stable in gastric cancer cells (Figures 1C,D), it is of great value to examine the expression pattern of circ0007360 in gastric cancer patient samples investigate whether *circ0007360* can be utilized as a biomarker for gastric cancer diagnosis in the future. To pave the way towards the clinical application of *circ0007360*, transcriptome data from patients with gastric cancer can be mined to test the correlation between *circ0007360* levels and clinicopathological parameters such as cancer stages and subtypes. Moreover, the patient survival data may provide insight into the prognostic significance of *circ0007360*.

*MiR-762* has been reported to be a tumor-promoting miRNA in multiple cancers, such as non-small cell lung cancer and breast cancer (Xu et al., 2021; Tokar et al., 2018; Huang et al., 2020). Although *circLPAR1* has been shown to harbor *miR-762* by sequence matching in bladder cancer cells, the biological significance of *miR-762* in regulating the effects mediated by *circLPAR1* in this setting has not been well studied. In our research, *circ0007630* is identified, for the first time, as another key upstream sponge for negatively controlling the endogenous levels of *miR-762* (Figure 2). Importantly, we consolidates the importance of *miR-762* mitigation for accomplishing the tumor-inhibitory role of *circ0007360* (Figure 6) and further links *IRF7* as a downstream effector of *miR-762* (Figures 7, 8). The latter also validates the results from a previous study on breast cancer, which demonstrated that *IRF7* is a key target mRNA of *miR-762* (Huang et al., 2020).

Recently, several research groups have shown that circRNAs are capable of encoding polypeptides that serve as scaffolds for certain proteins to exert their regulatory effects (Li et al., 2021; Liu et al., 2021; Shen et al., 2021). We examined the protein coding potential of the *bone fide* polypeptides of *circ0007360* and found that the coding probabilities of them are rather low (data not shown). Yet, we cannot rule out the possibility that polypeptides may be generated from *circ0007360* and contribute to the effects exerted by *circ0007360*. In the future investigation, proteome data can be mined to unravel whether polypeptides might be derived from circ0007360.

IRF7 belongs to the interferon regulatory factor family of transcription factors (Ning et al., 2011). In addition to its master role in controlling type I interferon production and immune system regulation, IRF7 acts as an oncogene or tumor suppressor, depending on the cell types used for investigations (Zhang et al., 2004; Andrews et al., 2002). Although the promoter of *IRF7* was hypermethylated in gastric cancer samples (Jee et al., 2009), the effect of IRF7 on gastric cancer progression has not been reported. In our study, through loss-of-function experiments, we find that IRF7 acts as a suppressor of the survival, migration, and invasion of gastric cancer cells (Figure 8 and Supplementary Figure S1). This may be further complemented by ectopic expression analysis to better understand the effects of IRF7 on gastric cancer progression. More importantly, we have shown that IRF7 is a negative regulator of stemness in gastric cancer cells. To uncover the mechanism by which IRF7 regulates gastric cancer cell stemness, pathways involved in stemness modulation, such as Wnt/β-catenin signaling (Zhan et al., 2017), can be detected and transcriptomic analysis upon IRF7 misexpression may aid in identifying the transactional target of this protein.

Although we have shown that the *circ0007360*/*miR-762/IRF7* axis is of pivotal significance in gastric cancer progression through *in vitro* and *in vivo* models, the correlations between these molecules can be checked in clinical gastric samples by analyzing the patient-derived RNA-seq data or by applying the detection on tissue microarrays from our patient cohort. These in-depth analyses will contribute to unveiling the sophisticated mechanisms of gastric cancer progression and shed light on the therapeutic benefits for patients with gastric cancer.

## Data Availability

The original contributions presented in the study are included in the article/Supplementary Material, further inquiries can be directed to the corresponding author.
