# Supplementary material for: Circular RNA circ0007360 Attenuates Gastric Cancer Progression by Altering the miR-762/IRF7 Axis
Source: Front Cell Dev Biol. 2022 Feb 17;10:789073. doi: 10.3389/fcell.2022.789073 (PMC8891931; doi:10.3389/fcell.2022.789073)
Supplement: Supplementary file 1 [file DataSheet1.docx]

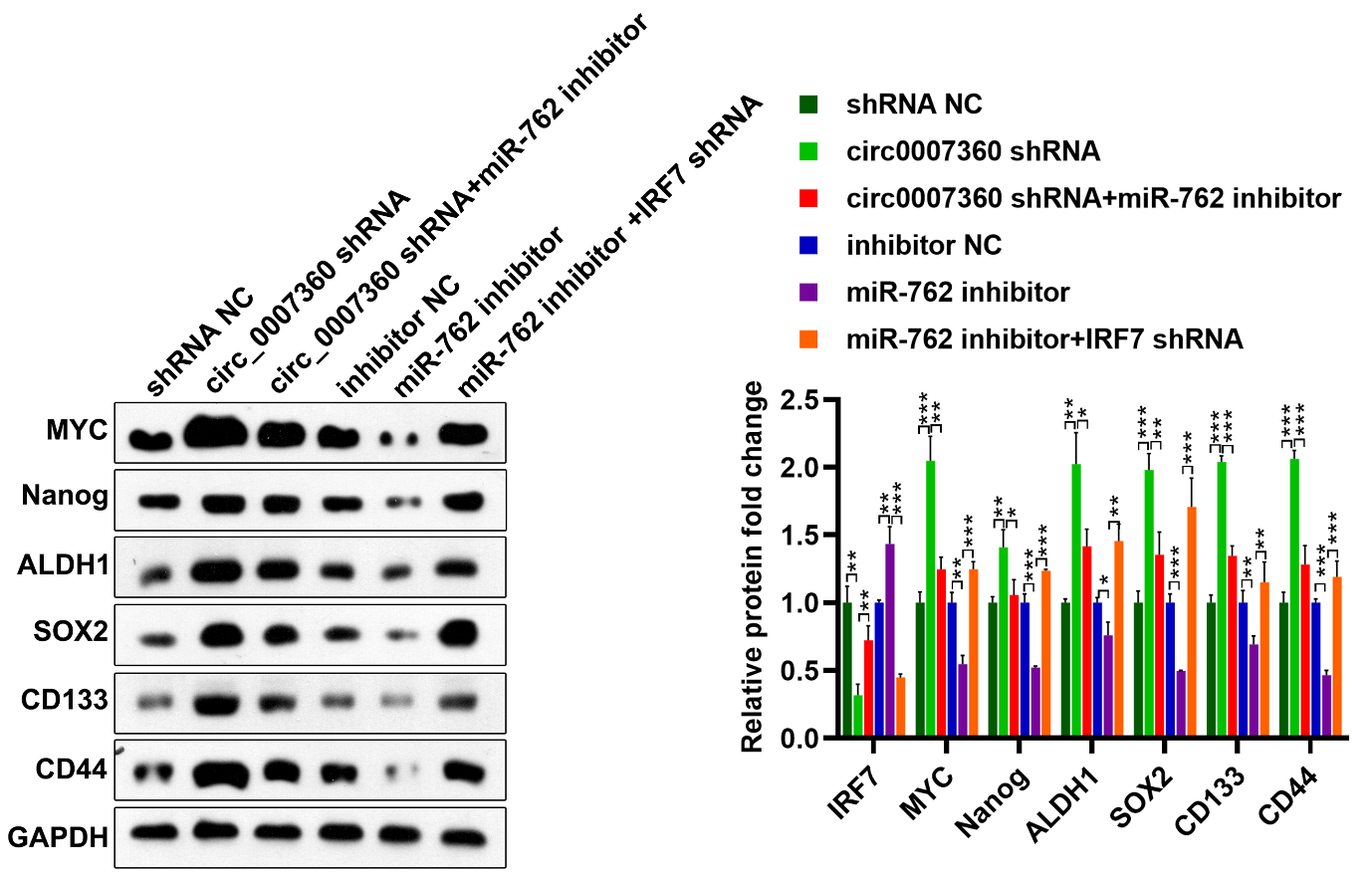


**Supplementary Figure 1.** The *circ0007360/miR-762/IRF7* axis alleviates the expression of stemness markers. Representative images (left) and quantification (right) of western blotting detection of markers involved in cell stemness regulation in AGS cells with indicated misexpression of *circ0007360*, *miR-762,* or *IRF7*.

**Supplementary Table 1. Primers for plasmid construction and RT-qPCR**

| Primers | | Sequence (5’-3’) |
| --- | --- | --- |
| *18S* | F | CGACGACCCATTCGAACGTCT |
|  | R | CTCTCCGGAATCGAA CCCTGA |
| *U6* | RT | CGCTTCACGAATTTGCGTGTCA |
|  | F | GCTTCGGCAGCACATATACTAAAAT |
|  | R | CGCTTCACGAATTTGCGTGTCAT |
| *Circ0007360* | F | GCGAATAGCCCAGTGAATG |
|  | R | GCAGCTCATCAGTGTCTGTG |
| *IRF7* | F | CCACGCTATACCATCTACCTGG |
|  | R | GCTGCTATCCAGGGAAGACACA |
| *miR-762* | RT | GTCGTATCCAGTGCAGGGTCCGAGGTATTCGCACTGGATACGACGCTCTG |
|  | F | GGGGCTGGGGCCGGGA |
|  | R | AGTGCAGGGTCCGAGGTATT |
| *IRF7* 3’UTR reporter | F | TCGAGAGAACTCCAGAAAGCTGGAGCAGCCCACCTAGAGCTGGCCGCGGCCG |
|  | R | TCGACCGGCCGCGGCCAGCTCTAGGTGGGCTGCTCCAGCTTTCTGGAGTTCT |
|  | Mut-F | TCGAGAGAACTCCAGAAAGCTGGAGGTCGGGTCCTAGAGCTGGCCGCGGCCG |
|  | Mut-R | TCGACCGGCCGCGGCCAGCTCTAGGACCCGACCTCCAGCTTTCTGGAGTTCT |
| *Circ0007360* reporter | F | GCTCGCTAGCCTCGAGATTATTCAGACAGTACCAAC |
|  | R | ATGCCTGCAGGTCGACATCTGAACCGACATTTCTCATG |
|  | Mut-F | TGTGGTAGGTCGGGCTTCAGCGAATAGCCCAGTG |
|  | Mut-R | CTGAAGCCCGACCTACCACATTCACCACGTGATTAG |
| *Circ0007360* OE | F | ACTTTTTTTTTATACTTCAGATTCTGCTCTCTTTGCTGAAC |
|  | R | TTCTTTTCCTTGCTTCTTACATCTGAACCGACATTTCTC |
| *IRF7* sh1 | F | CCGGCCTGCTCCAGGCAGTGCAACACTCGAGTGTTGCACTGCCTGGAGCAGGTTTTT |
|  | R | AATTAAAAACCTGCTCCAGGCAGTGCAACACTCGAGTGTTGCACTGCCTGGAGCAGG |
| *IRF7* sh2 | F | CCGGCGAGCTGCACGTTCCTATACGCTCGAGCGTATAGGAACGTGCAGCTCGTTTTT |
|  | R | AATTAAAAACGAGCTGCACGTTCCTATACGCTCGAGCGTATAGGAACGTGCAGCTCG |
| *IRF7* sh3 | F | CCGGGGTGTGTCTTCCCTGGATAGCCTCGAGGCTATCCAGGGAAGACACACCTTTTT |
|  | R | AATTAAAAAGGTGTGTCTTCCCTGGATAGCCTCGAGGCTATCCAGGGAAGACACACC |
| *Circ0007360* sh1 | F | CCGGGGTTCAGATATTCTGCTCTCTCTCGAGAGAGAGCAGAATATCTGAACCTTTTT |
|  | R | AATTAAAAAGGTTCAGATATTCTGCTCTCTCTCGAGAGAGAGCAGAATATCTGAACC |
| *Circ0007360* shNC1 | F | CCGGCCAAGTCTAATTCTGCTCTCTCTCGAGAGAGAGCAGAATTAGACTTGGTTTTT |
|  | R | AATTAAAAACCAAGTCTAATTCTGCTCTCTCTCGAGAGAGAGCAGAATTAGACTTGG |
| *Circ0007360* sh2 | F | CCGGAAATGTCGGTTCAGATATTCTCTCGAGAGAATATCTGAACCGACATTTTTTTT |
|  | R | AATTAAAAAAAATGTCGGTTCAGATATTCTCTCGAGAGAATATCTGAACCGACATTT |
| *Circ0007360* shNC2 | F | CCGGAAATGTCGGTTCAGATTAAGACTCGAGTCTTAATCTGAACCGACATTTTTTTT |
|  | R | AATTAAAAAAAATGTCGGTTCAGATTAAGACTCGAGTCTTAATCTGAACCGACATTT |

**Supplementary Table 2. Antibodies for western blotting**

| **Antibodies** | **Provider** | **Catalog NO.** | **Dilution** |
| --- | --- | --- | --- |
| GAPDH | Proteintech, Wuhan, China | 10494-1-AP | 1:3000 |
| IRF7 | Proteintech, Wuhan, China | 22392-1-AP | 1:2000 |
| Myc | Proteintech, Wuhan, China | 67447-1-Ig | 1:10000 |
| Nanog | Proteintech, Wuhan, China | 14295-1-AP | 1:1000 |
| ALDH1 | Proteintech, Wuhan, China | 15910-1-AP | 1:3000 |
| SOX2 | Proteintech, Wuhan, China | 11064-1-AP | 1:1000 |
| CD133 | Proteintech, Wuhan, China | 18470-1-AP | 1:1500 |
| CD44 | Proteintech, Wuhan, China | 15675-1-AP | 1:5000 |
| HRP Goat Anti-mouse IgG(H+L) | Proteintech, Wuhan, China | SA00001-1 | 1:10000 |
| HRP Goat Anti-Rabbit IgG(H+L) | Proteintech, Wuhan, China | SA00001-2 | 1:10000 |
